# Structural modeling of human AKAP3 protein and in silico analysis of single nucleotide polymorphisms associated with sperm motility

**DOI:** 10.1038/s41598-022-07513-9

**Published:** 2022-03-07

**Authors:** Alemeh Rafaee, Elaheh Kashani-Amin, Anahita Mohseni Meybodi, Azadeh Ebrahim-Habibi, Marjan Sabbaghian

**Affiliations:** 1 Department of Andrology, Reproductive Biomedicine Research Center, Royan Institute for Reproductive Biomedicine, ACECR, Tehran, Iran; 2 Department of Biology, Science and Research Branch, Islamic Azad University, Tehran, Iran; 3 Biosensor Research Center, Endocrinology and Metabolism Molecular-Cellular Sciences Institute, Tehran University of Medical Sciences, Tehran, Iran; 4 Department of Genetics, Reproductive Biomedicine Research Center, Royan Institute for Reproductive Biomedicine, ACECR, Tehran, Iran; 5 Department of Pathology and Laboratory Medicine, Western University, London, Ontario Canada

**Keywords:** Computational biology and bioinformatics, Genetics

## Abstract

AKAP3 is a member of the A-kinase anchoring proteins and it is a constituent of the sperm fibrous sheath. AKAP3 is needed for the formation of sperm flagellum structure, sperm motility, and male fertility. This study aims to model the AKAP3 tertiary structure and identify the probable impact of four mutations characterized in infertile men on the AKAP3 structure. The T464S, I500T, E525K, and I661T substitutions were analyzed using in silico methods. The secondary structure and three-dimensional model of AKAP3 were determined using PSI-BLAST based secondary structure prediction and Robetta servers. The TM-score was used to quantitatively measure the structural similarities between native and mutated models. All of the desired substitutions were classified as benign. I-Mutant results showed all of the substitutions decreased AKAP3 stability; however, the I500T and I661T were more effective. Superposition and secondary structure comparisons between native and mutants showed no dramatic deviations. Our study provided an appropriate model for AKAP3. Destabilization of AKAP3 caused by these substitutions did not appear to induce structural disturbances. As AKAP3 is involved in male infertility, providing more structural insights and the impact of mutations that cause protein functional diversity could elucidate the etiology of male fertility problems at molecular level.

## Introduction

Motility is one of the most peculiar functions of mature spermatozoa. Regulation of this process is controlled by a complex balance between kinase and phosphatase enzymes that permits the spermatozoa to swim^1,2^. The cyclic adenosine monophosphate/protein kinase A (cAMP/PKA) dependent pathway plays an important role in tyrosine phosphorylation of sperm flagellar proteins and it can lead to an increase in sperm motility^3^. These processes are under the control of numerous factors, including bicarbonate (HCO_3_^−^). HCO_3_^−^ primarily stimulates soluble adenylate cyclase (sAC), which leads to an increase in cAMP concentration and subsequently results in tyrosine phosphorylation of A-kinase anchoring protein 3 (AKAP3) and cAMP-dependent protein kinase A (PKA) recruitment^4^.

A-kinase anchoring proteins (AKAPs) are a group of signal-organizing scaffolding proteins implicated in various cellular functions by anchoring PKA; therefore, they assemble multi-protein signaling complexes to integrate cAMP signaling with other pathways and signaling events^5^.

AKAP3 is also known as AKAP110, cancer/testis antigen 82 (CT82), and fibrous sheath protein of 95 kDa (FSP95). It is a testis-specific gene expressed in spermatids and mature spermatozoa (https://www.uniprot.org/)^6^. AKAP3, in conjunction with AKAP4, are the major constituents of the sperm tail fibrous sheath; their coordination is crucial for the organization of different enzymes into a signaling platform in the fibrous sheath that supports sperm motility^7,8^. Phosphorylation of AKAP3, selective recruitment, and an increase in PKA and AKAP3 binding in human spermatozoa eventually stimulate sperm motility; however, the role of AKAP3 gene expression on PKA function is still confusing due to the lack of structural information during spermiogenesis^8,9^.

The AKAP3 protein consists of 853 amino acids and has a molecular weight of approximately 95 kDa (https://www.uniprot.org/). AKAP3 has two helical regions located in the N-terminal end (amino acids 124–143 and 278–300 in mice) that are constructed from two amphipathic peptides. The distribution of hydrophobic amino acids along one side and hydrophilic amino acids on the other side of the helices provide a secondary structure that allows the AKAP3 to interact with the PKA regulatory subunits^7^. SPA17, ROPN1, and CABYR are other proteins that interact with AKAP3 via the PKA binding site^10,11^. AKAP3 also contains a C-terminal domain, named AKAP110 (amino acids 166–853), that is responsible for interaction with AKAP4 (amino acids 488–583) and Gα13 (amino acids 685–853)^12,13^. Activated Gα13 binds to AKAP3 and induces the release of the catalytic subunit from the regulatory subunit of PKA. This results in cAMP-independent activation of PKA. So Gα13-AKAP3 interaction could be associated with regulation of sperm motility^13^. AKAP3 null spermatozoa have morphological abnormalities, disruptions in the fibrous sheath, lack of subcellular structures, misregulation of PKA signaling, and mislocalizations of some proteins^14^.

Nonsynonymous single nucleotide polymorphisms (nsSNPs) are a type of SNP that cause alterations in the corresponding protein amino acid sequence. nsSNPs might exert a detrimental impact on protein properties including structure, function, and stability, and consequently lead to phenotypic effects^15,16^. The amino acid changes are likely to induce deleterious structural influences on the native protein conformation. Thus, they account for changes in time-dependent physiological affinities of proteins and in the biochemical pathways^17^.

An in-depth study of the genetic mutations and their molecular basis for invoking disease-related pathways is an important part of genomic research^18^. Recently, in silico approaches have been extensively applied to determine the likely influences of deleterious nsSNPs on both structure and function of candidate proteins by taking into consideration the conservation of sequences across species, structural attributes, and physicochemical properties of polypeptides^19^. The ability to distinguish pathogenic and neutral nsSNPs by computational methods could significantly aid in targeting disease-associated mutations by filtering out the most likely pathological variations from a large pool of SNP datasets. Therefore, it will benefit the foundation of genome-level studies, and establish future insights for target based therapies and personalized medicine^20^. In comparison to laboratory-based characterizations, benefits of computational methods include convenience, reliability, speed, and also lower cost for assessment the effects of SNPs^19^.

Our unpublished findings in patients with immotile short tail sperm defect (ISTS) and oligoasthenoteratozoospermia showed some variations in the AKAP3 gene. Some of these variants are located within the AKAP3 binding site for AKAP4. Therefore, we developed the concept of this study based on the results of our previous investigation and the causative role of AKAP3 in sperm motility. This project aimed to construct a three-dimensional (3D) model of AKAP3 and use in silico approaches to predict the probable impact of characterized amino acid substitutions on the AKAP3 structure. The results provide 3D structural information on the AKAP3 protein as a gene responsible for male infertility; in addition, it enables future investigations to target disease-associated mutations with more confidence and obtain more structural data about the etiology of male infertility.

## Results

### SNPs information

Mutational analysis of the AKAP3 gene disclosed six SNPs located within exon 5. Table 1 summarizes the SNP information retrieved from the dbSNP database. Among the identified SNPs, two were synonymous (rs10774251 and rs11063265) and the remaining four were missense variations (rs11063266, rs12366671, rs1990312, and rs1990313), which we considered for further computational analysis.

**Table 1:** List of identified single nucleotide polymorphisms (SNPs) and their corresponding amino acid substitutions.

Variation	dbSNP ID	Wild type amino acid	Mutant amino acid	Position	Variation type
1378T>C	rs10774251	L	L	460	Synonymous
1391C>G	rs11063266	T	S	464	Missense
1437T>C	rs11063265	F	F	479	Synonymous
1499T>C	rs12366671	I	T	500	Missense
1573G>A	rs1990312	E	K	525	Missense
1982T>C	rs1990313	I	T	661	Missense

### ConSurf results detected evolutionarily conserved regions

The ConSurf web-server was used to determine the evolutionarily conserved regions and amino acids of AKAP3. The AKAP3 FASTA sequence was submitted to ConSurf and the conservation scores were determined based on multiple sequence alignment of 66 homologs.

**Figure 1:**
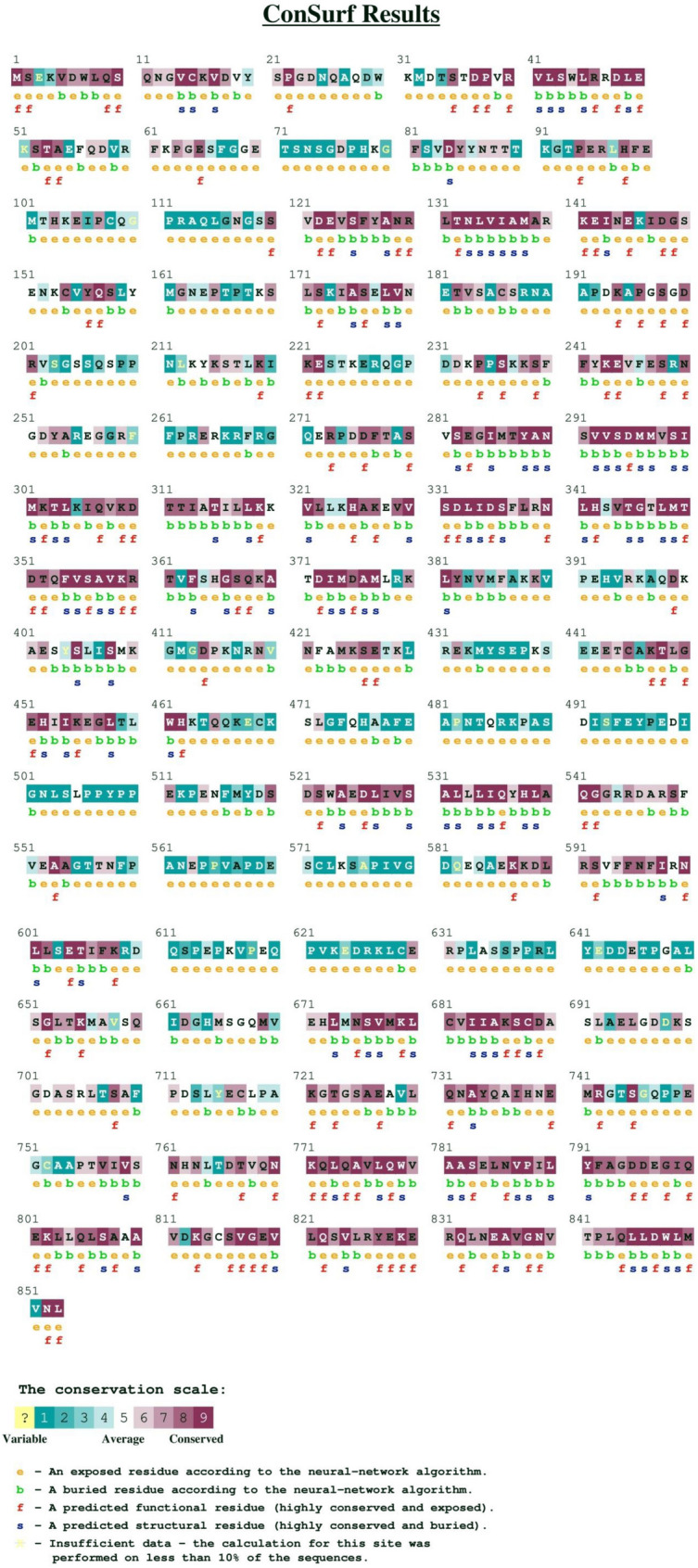
ConSurf results for detection of evolutionarily conserved regions in AKAP3 protein. Amino acids were ranked and highlighted on a conservation scale of 1–9.

The ConSurf results classified T464, I500, and I661 as “variable” residues with a conservation score of 1. E525 was predicted to be a conserved amino acid that had a conservation score of 7. T464, I500, and E525 were exposed residues and I661 was buried. None of the amino acids were characterized as functional or structural residues (Fig. 1). Residues 122–145 and 273–377 in the AKAP3 structure were extensively more conserved in comparison with other regions. Approximately 180 of the last amino acids at the C-terminal were highly conserved, which indicated that this area has a crucial functional or structural role in AKAP3.

### In silico analysis results of nsSNPs

Bioinformatics prediction tools revealed that all four nsSNPs were benign variants (Table 2). Evolutionary analysis of coding SNPs using the PANTHER server also predicted that all of the substitutions were “probably benign” and the probability of deleterious effects for T464S and E525K were 0.02, whereas it was 0.19 for I500T and I661T.

**Table 2:** Prediction of the impact of nonsynonymous single nucleotide polymorphisms (nsSNPs) on proteins by using bioinformatics tools.

dbSNP ID	Amino acid change	SIFT score	SIFT prediction	PolyPhen-2 score	PolyPhen-2 prediction	PROVEAN score	PROVEAN prediction	MutPred score	MutPred prediction
rs11063266	T464S	1.000	Tolerated	0.000	Benign	0.217	Neutral	0.036	–
rs12366671	I500T	0.420	Tolerated	0.000	Benign	1.366	Neutral	0.038	–
rs1990312	E525K	1.000	Tolerated	0.000	Benign	1.305	Neutral	0.074	–
rs19990313	I661T	0.080	Tolerated	0.068	Benign	0.269	Neutral	0.151	–

*SIFT* Sorting Intolerant from Tolerant server v.6.2.1, *PolyPhen-2* Polymorphism Phenotyping v2, *PROVEAN* Protein Variation Effect Analyzer v.1.1

### Comparison of wild type and mutant amino acid properties using HOPE results

Project HOPE was used to evaluate the wild type and mutant amino acid differences in terms of specific size, charge, hydrophobicity-value, and probable interactions that might be induced by mutated residues. For the T to S conversion at position 464, the mutated residue was smaller than the wild type T and might result in loss of interactions. Replacement of I (nonpolar) with T (polar) at amino acid positions 500 and 661 might disrupt hydrophobic interactions, either in the core of the protein or on the surface. In terms of the E525K substitution, the wild type and mutant residues differed in size and charge. In this mutation, the negatively charged small residue was replaced by a positively charged large moiety that could cause repulsion with other residues in the protein or ligands. Additionally, the difference in size of lysine compared to glutamic acid might create bumps in the protein. If the substituting amino acid does not fit into the protein, it causes structural alterations that are sometimes harmful. Clashes of amino acids lead to local or global structural changes. For structural and physical reasons, the amino acid size and side-chain conformations must be structurally able to fit into the new position.

### I-Mutant and MUpro scores predicted protein stability changes upon mutations

Four nsSNPs were submitted to the I-Mutant server to calculate ΔΔ *G* and the reliability index (RI). Based on I-Mutant scores, all identified amino acid changes were predicted to decrease the stability of the AKAP3 protein. I500T and I661T seemed to be more effective with a ΔΔ *G* of − 4.42, and − 2.15, respectively (Table 3). The MUpro tool results were consistent with the I-Mutant server, and all amino acid changes led to decreased protein stability. The confidence score for T464S was − 0.63929138 and it was − 1 for the rest of the substitutions.

**Table 3:** I-Mutant output for protein stability changes upon mutations. Stability changes were calculated at pH 7 and 25 °C. *ΔΔG Value* ΔG (New Protein) − ΔG (Wild Type) in Kcal/mol, *ΔΔG < 0* decreased stability, *ΔΔG > 0* increased stability, *RI* reliability index, *ΔΔG* Gibbs-free energy change.

dbSNP ID	Amino acid change	pH	Temperature (°C)	Stability	RI	DDG (kcal/mol)
rs11063266	T464S	7.0	25	Decrease	3	− 0.78
rs12366671	I500T	7.0	25	Decrease	9	− 4.42
rs1990312	E525K	7.0	25	Decrease	1	− 0.75
rs19990313	I661T	7.0	25	Decrease	7	− 2.15

### Homology modeling, validation, and quality estimation of the native and mutated models of AKAP3

**Figure 2:**
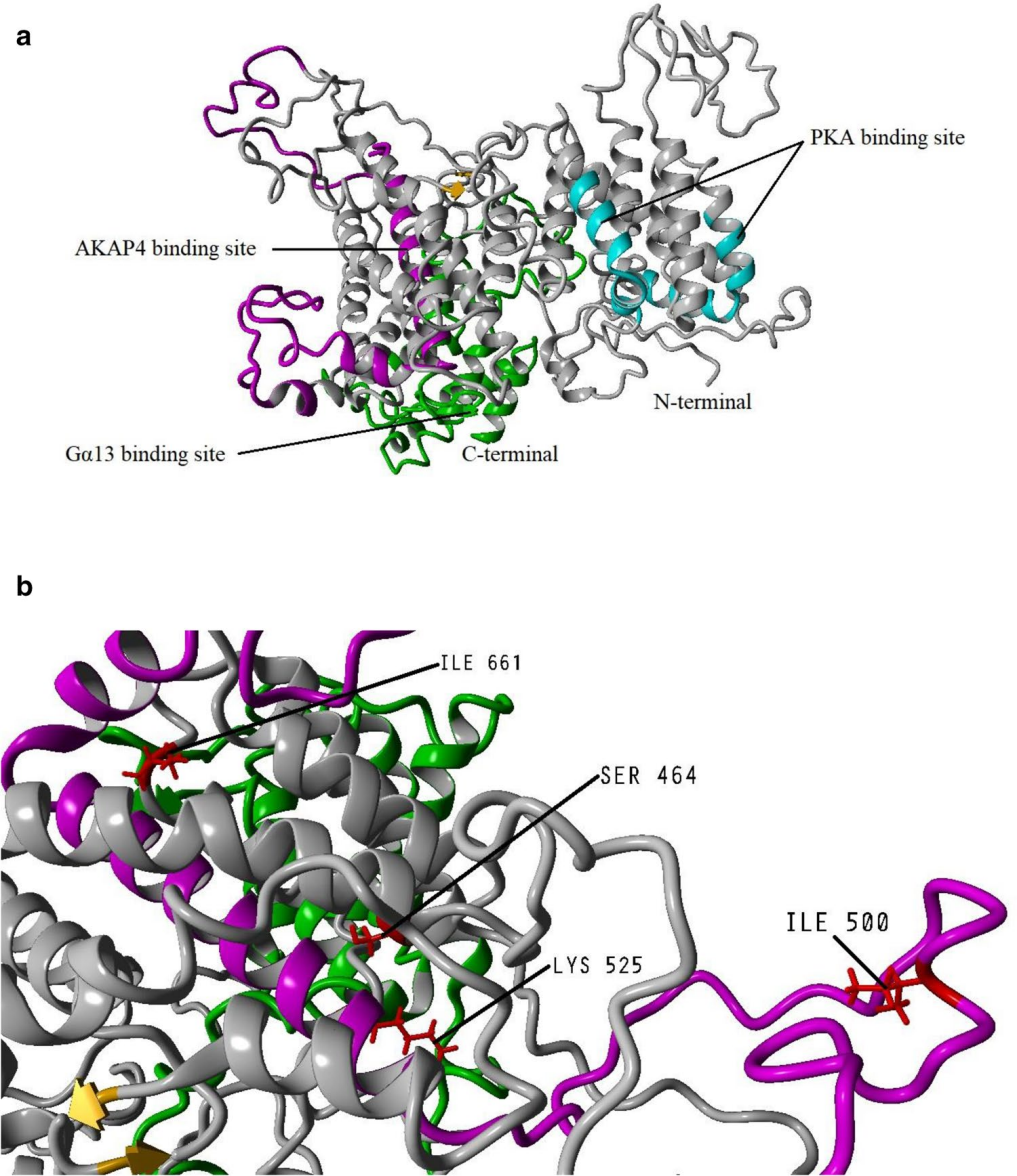
Cartoon representation of the AKAP3 predicted model. (**a**) The model was developed by the Robetta server and visualized using YASARA software v.20.7.4 (http://www.yasara.org/index.html). The binding sites for protein kinase A (PKA), Gα13, and AKAP4 are shown in cyan, green, and magenta respectively. (**b**) Deep view of the locations of the important amino acids detected in our population.

To analyze the damaging impact of I500T and I661T at the structural level, the full-length AKAP3 (853 amino acids) tertiary structure was modeled by Robetta comparative modeling approach. Both T464S and E525K were characterized in all of the studied individuals; therefore, these mutations were inserted into AKAP3 FASTA and the obtained model was considered the major AKAP3 structure in our population. Robetta used the templates below for model prediction: 1wvtA, 2r6tA, 3uekA, 3w3vA, 3wyfF, 4oo6A, 5disA, 5ve8A, 5wtjA, 5wtjB, 5wtkA, 5yfpD, 6em8A, 6hmkA, 6ig9T, 6o9yA, 6oa0A, 6oa3A, 6tnfA, 6tnfB, 6tvoA, 6v85A, 6v86A, 6x2mC, 6x2oC, 6x2wC, and 6x2yC. In order to improve the accuracy of the constructed 3D model, energy minimization was carried out with the YASARA energy minimization server, YASARA force field (http://www.yasara.org/minimizationserver.htm). Figure 2 shows the 3D visualization of the AKAP3 structure.

**Figure 3:**
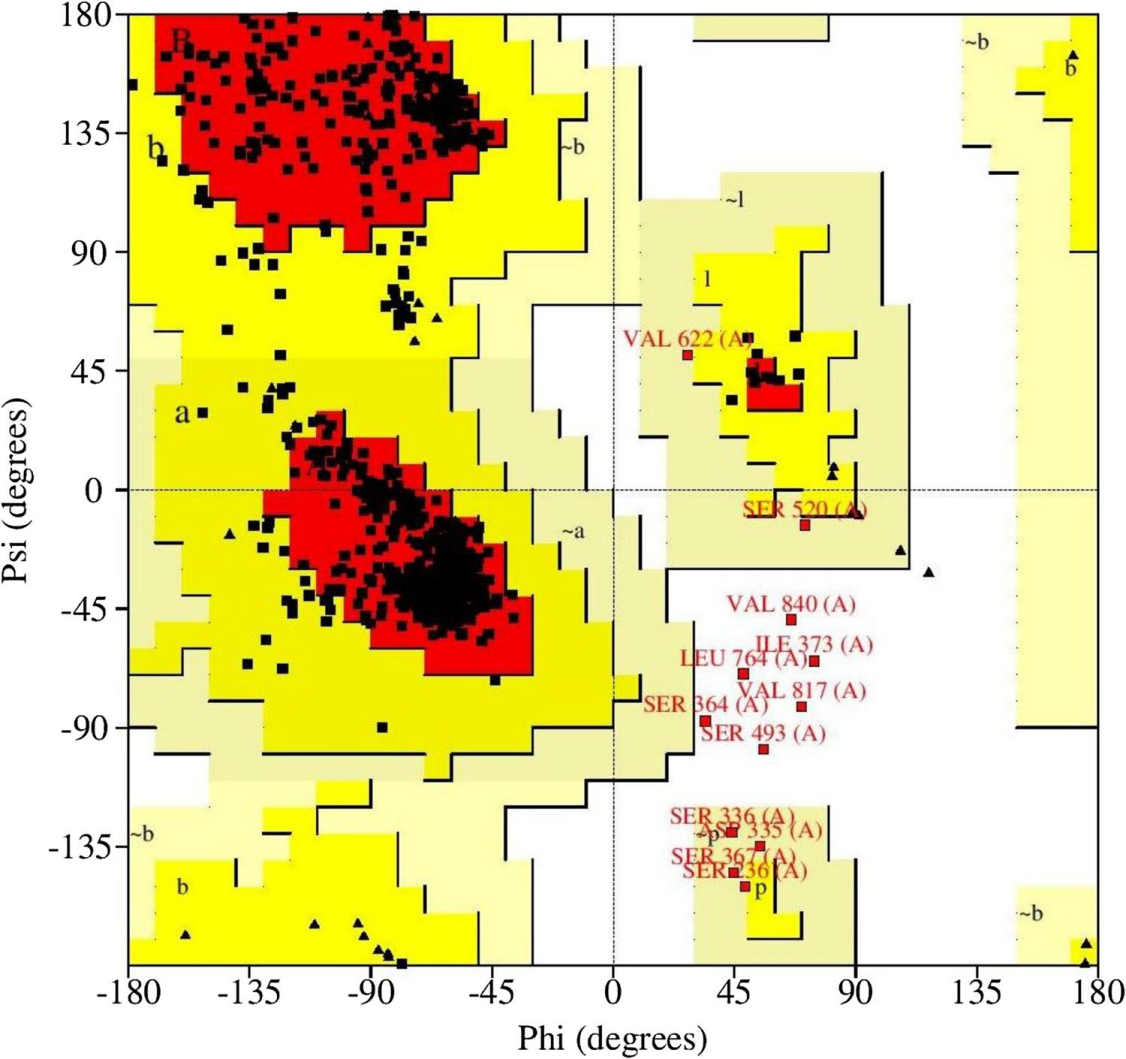
POROCHECK-Ramachandran plot of the AKAP3 predicted model. The most favored, additional allowed, generously allowed, and disallowed regions are colored in red, yellow, light yellow, and white respectively.

**Figure 4:**
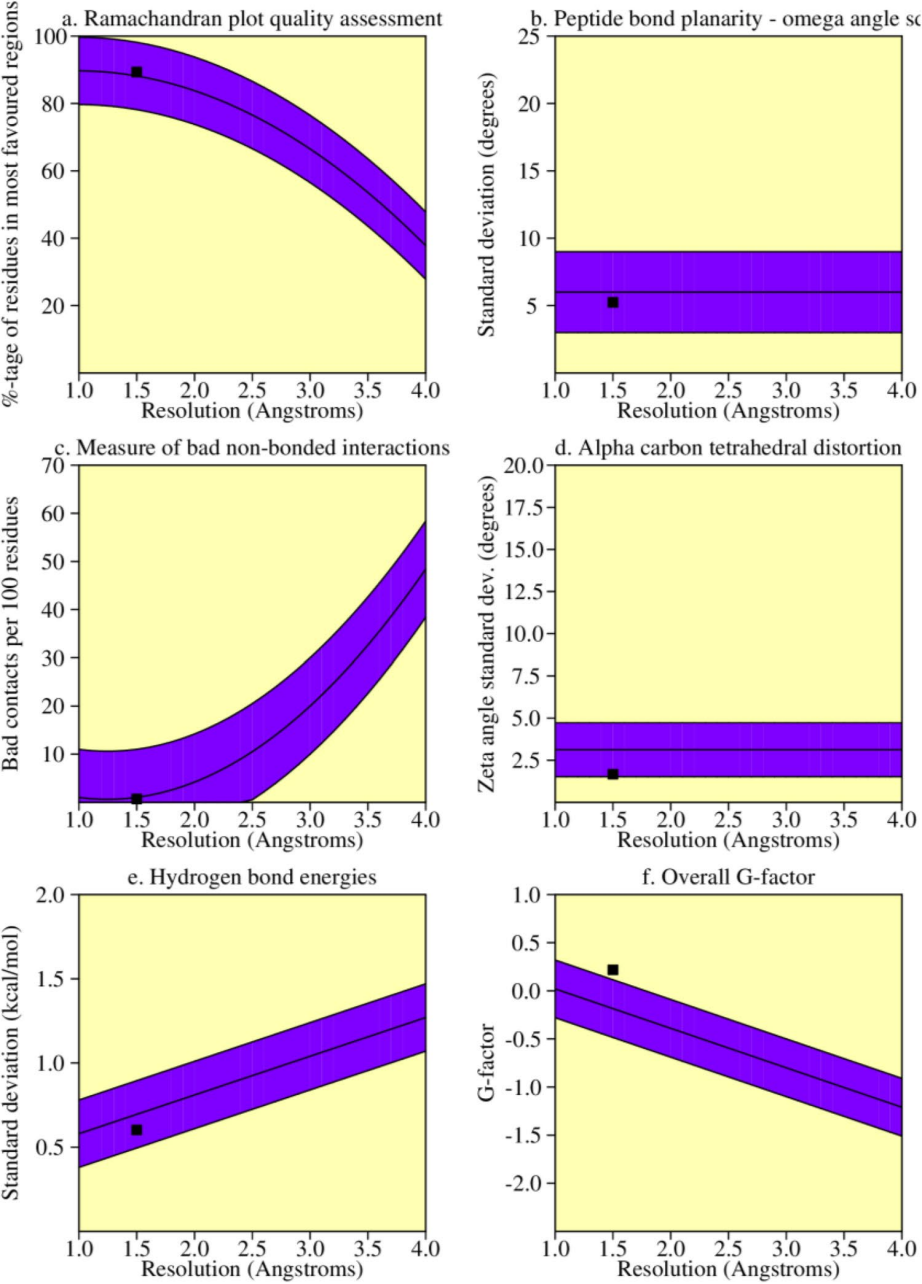
The main-chain parameters of AKAP3 modeled structure. The plots are generated by PROCHECK v.3.0.

**Figure 5:**
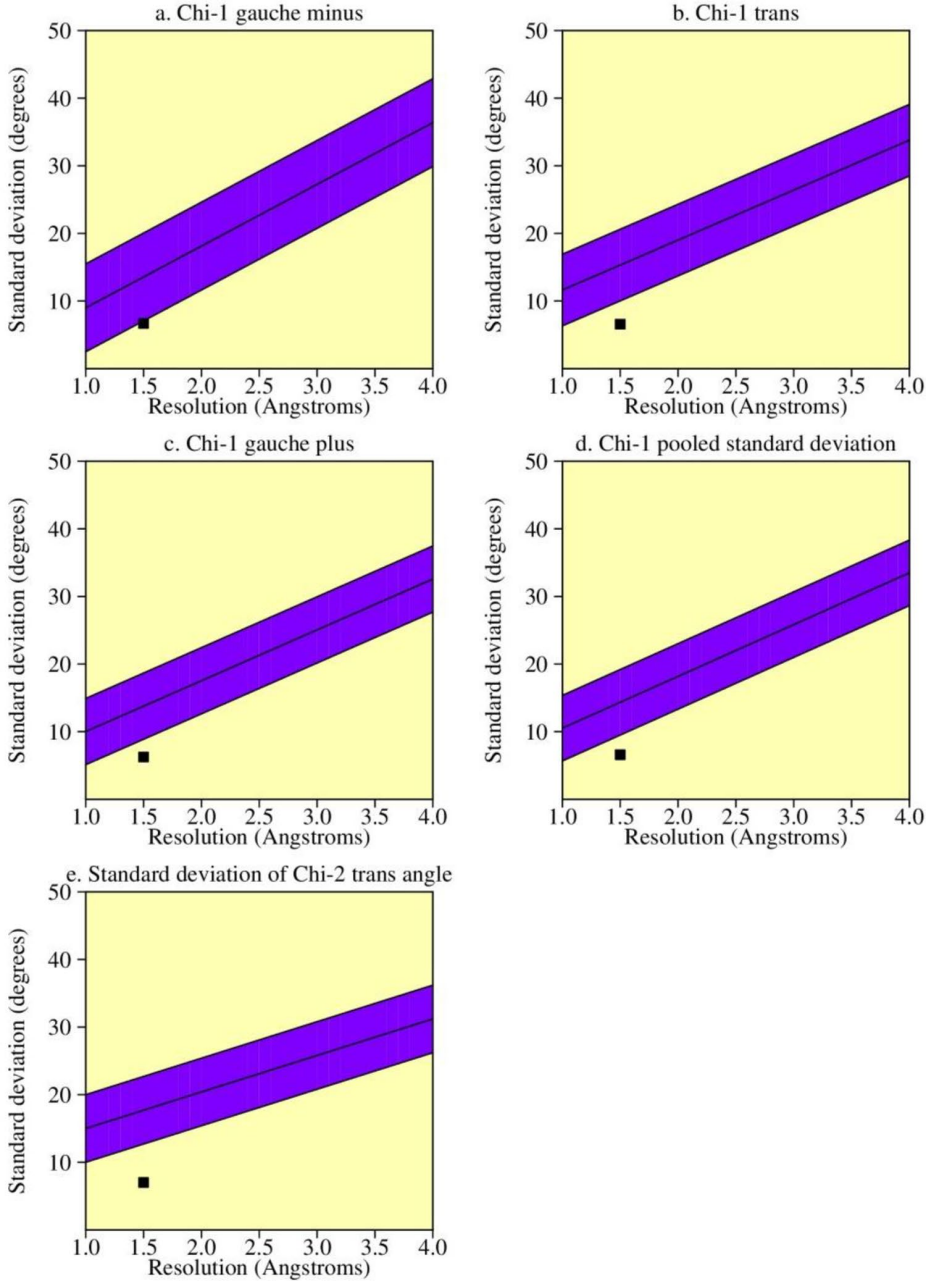
The side-chain parameters of AKAP3 modeled structure. The plots are generated by PROCHECK v.3.0.

**Figure 6:**
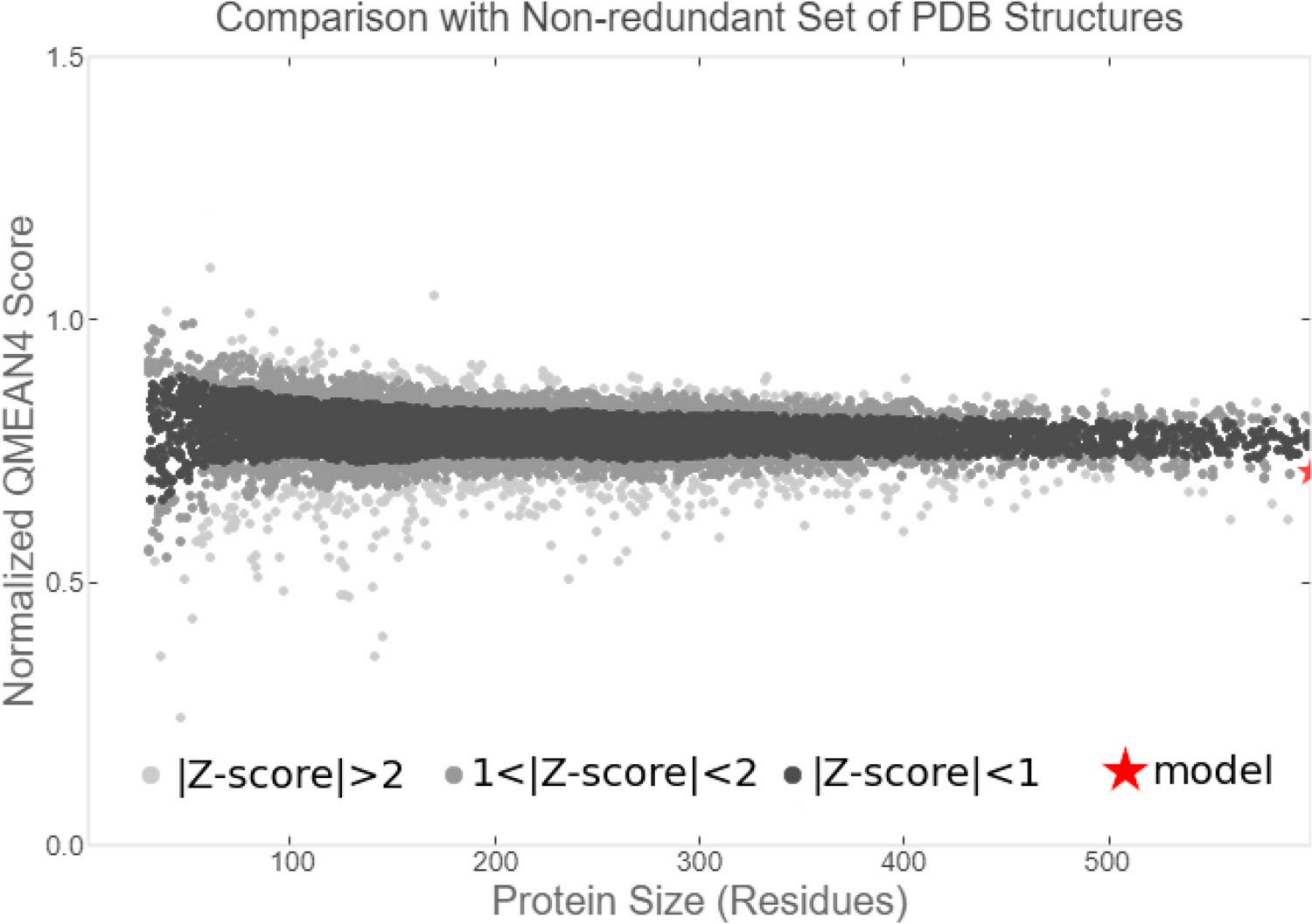
Comparison plot indicates the quality of the model in comparison with experimental structures of similar sizes. The *x*-axis shows the protein length. The *y*-axis is the normalized QMEAN score. The AKAP3 predicted model is represented as a red star.

The AKAP3 energy minimized model was validated by the PROCHECK-Ramachandran plot. The results demonstrated that 672 out of 853 (89.4%) amino acids were located in the most favored regions; 68 (9.0%) in the additional allowed regions; 6 (0.8%) in the generously allowed regions; and 6 (0.8%) in the disallowed regions. The overall average of the G-factors was 0.25, which was favorable. A low or negative G-factor is an unfavorable parameter in model quality estimation, and values less than − 0.5 and − 1 are interpreted as unusual and very unusual, respectively. The model comprised of 752 (88.1%) non-glycine and non-proline residues, 50 glycine (shown as triangles) residues, 49 proline residues, and 2 end-residues (excl. Gly and Pro) (Fig. 3). Among the stereochemical parameters of the main chain, the overall G-factor represented better quality compared to the ideal values and the remaining five properties were inside the suitable regions (Fig. 4; Table 4). Besides, all of the side-chain stereochemical parameters also showed better quality in comparison with the ideal values (Fig. 5; Table 5). Model quality estimation by QMEAN provided a QMEAN Z-score of − 1.49. Figure 6 is a comparison plot that shows the AKAP3 model quality score in comparison with experimental structures of similar size. The 3D structures of the I500T and I661T mutated models were also constructed using the Robetta server and energy minimization was performed. Table 6 summarizes the validation results of the mutant models. Thus, the predicted structures can be considered to be appropriate and reliable models based on the Ramachandran plot and QMEAN score.

**Table 4:** Stereochemical parameters of the main-chain obtained from the PROCHECK program.

Stereochemical parameter	No. of data pts	Comparison values		Band width	No. of band widths from mean
		Parameter value	Typical value		
%-tage residues in A, B, L	752	89.4	88.2	10.0	0.1 Inside
Omega angle standard deviation	843	5.2	6.0	3.0	− 0.3 Inside
Bad contacts/100 residues	6	0.7	1.0	10.0	− 0.0 Inside
Zeta angle standard deviation	803	1.7	3.1	1.6	− 0.9 Inside
H-bond energy standard deviation	508	0.6	0.7	0.2	− 0.5 Inside
Overall G-factor	853	0.2	− 0.2	0.3	1.3 Better

**Table 5:** Stereochemical parameters of the side-chains obtained from the PROCHECK program.

Stereochemical parameter	No. of data pts	Comparison values		Band width	No. of band widths from mean
		Parameter value	Typical value		
Chi-1 gauche minus standard deviation	107	6.7	13.6	6.5	− 1.1 Better
Chi-1 trans standard deviation	199	6.6	15.3	5.3	− 1.6 Better
Chi-1 gauche plus standard deviation	387	6.2	13.8	4.9	− 1.5 Better
Chi-1 pooled standard deviation	693	6.6	14.3	4.8	− 1.6 Better
Chi-2 trans standard deviation	214	7.0	17.7	5.0	− 2.1 Better

**Table 6:** The PROCHECK validation results for both the I500T and I661T mutated three-dimensional (3D) structures.

Model name	G-factor	Most favored	Additional allowed	Generously allowed	Disallowed
Mutant-I500T	0.21	653 (86.8%)	89 (11.8%)	5 (0.7%)	5 (0.7%)
Mutant I661T	0.22	658 (87.5%)	88 (11.7%)	3 (0.4%)	3 (0.4%)

### Comparison between predicted secondary structures of AKAP3 using PSIPRED and DSSP

PSIPRED provided the secondary structure and distributions of the alpha helices, beta-sheets, and coils in the AKAP3 native structure. The coils were the dominant secondary structure elements (50.5%), followed by alpha helix (45.9%) and beta-sheets (3.5%) (Supplementary Fig. 1). DSSP server also assigned the secondary structures of the predicted models; accordingly, there were 83.6%, 82.5%, and 81.5% identities between PSIPRED predictions and DSSP outputs for the native, I500T, and I661T models, respectively.

### MD simulation experiment findings

**Figure 7:**
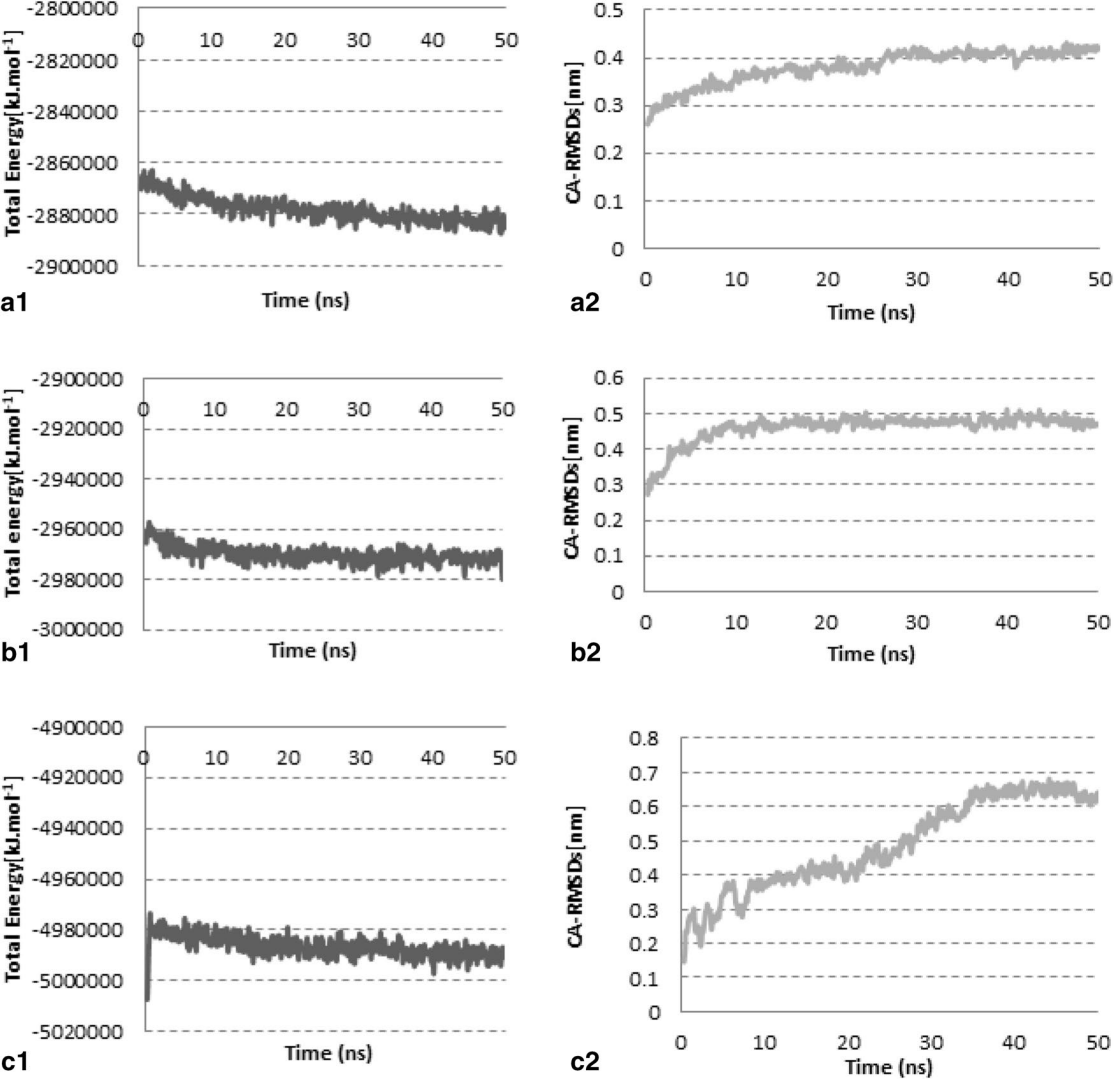
Investigation of the model stability based on total potential energy and CA-RMSD values. (**a1**,**a2**) Native, (**b1**,**b2**) I500T mutant, and (**c1**,**c2**) I600T mutant models**.**

MD simulation experiment results are shown in Fig. 7, and the average values for total potential energies and carbon-alpha RMSDs are presented in Table 7.

**Table 7:** The average values for total potential energies and CA-RMSDs of the three models during the 50 ns MD simulation time span. *CA-RMSDs* carbon-alpha root mean square deviations; values for total potential energies and CA-RMSDs, as well as the CA-RMSDs average of the last 10 ns are presented as ± standard deviation (SD).

Models	Average total potential energy (kJ·mol^−1^)	CA-RMSD average (nm)	The last 10 ns CA-RMSD average (nm)
Native form	− 2,878,196 ± 4620	0.38 ± 0.035	0.412 ± 0.010
Mutant-I500T	− 2,970,040 ± 3273	0.46 ± 0.038	0.483 ± 0.011
Mutant-I661T	− 4,986,408 ± 4134.5	0.49 ± 0.13	0.64 ± 0.16

#### Native model

The graphs in Fig. 7a1,a2 show that the system reached a steady state after 20 ns.

#### I500T mutant model

**Figure 8:**
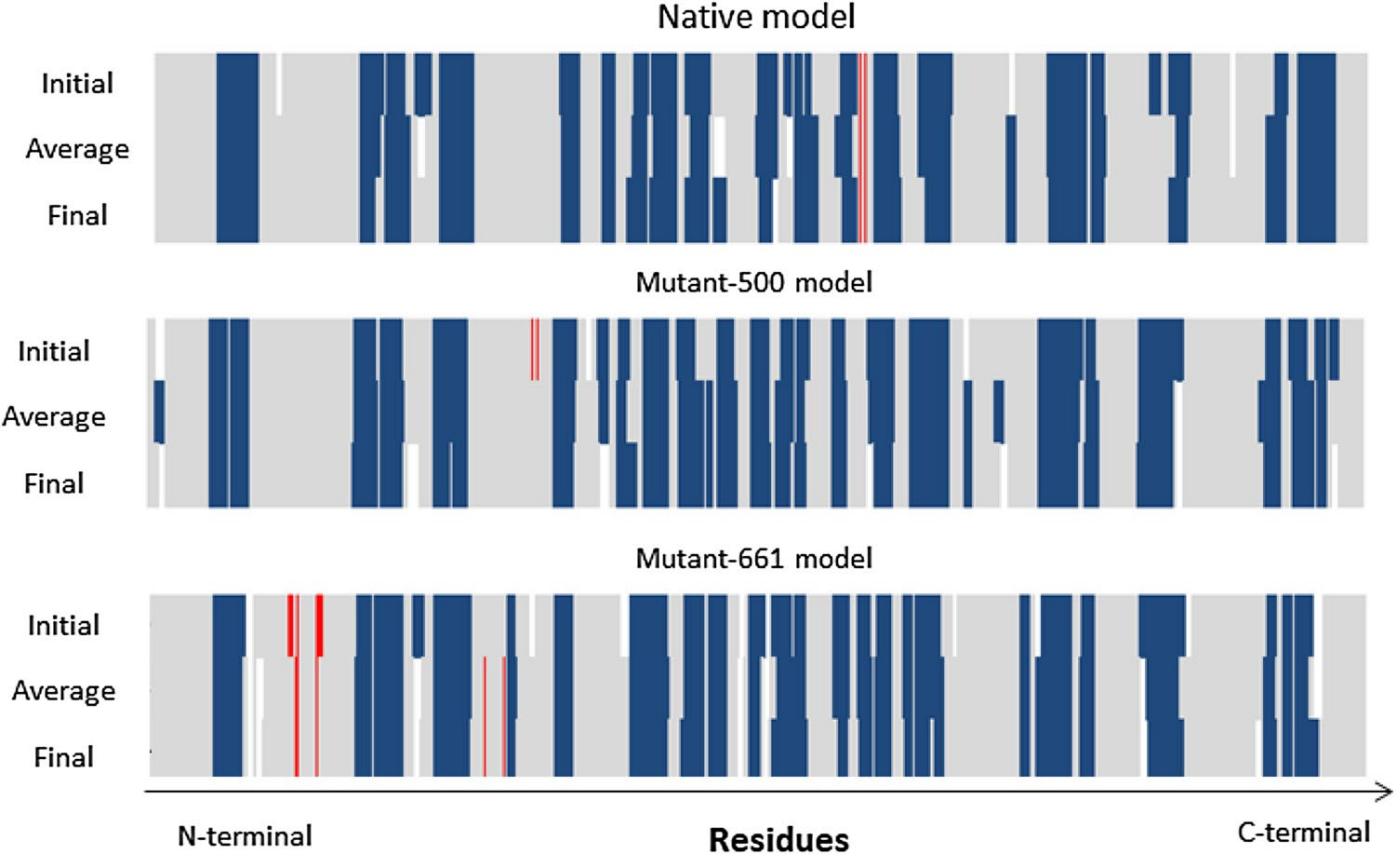
Investigation of the stability of local structures during the simulation time span. Secondary structures of the initial, average, and final conformations were depicted for each model. Blue, red, and grey colors represent alpha helices, beta-sheets, and coils (or turns), respectively.

Graphs in Fig. 7b1,b2 show that the model was stable after 20 ns. However, the energy level was higher than the native form, and CA-RMSDs were higher than the native form with more fluctuations. Table 7 shows that the average values for CA-RMSDs of native and mutant-I500T models were higher than the optimum value of 0.2 nm because the CA-RMSDs were calculated by taking into consideration the initial position. As shown in Fig. 7a2,b2, structure movements did not exceed 0.2 nm after the equilibration time. CA-RMSDs obtained by superposing the average conformation on the initial and final conformations of the native model were 0.4 nm and 0.15 nm, respectively. Residue-by-residue secondary structure analysis confirmed the stability of the local structures (Fig. 8). These expressions were true for the mutant-I500T model either; superposing average/initial and average/last conformations resulted in 0.48 nm and 0.14 nm, respectively. Residue-by-residue secondary structure analysis showed that local structures remained almost intact during the simulation time span (Fig. 8).

#### I661T mutant model

After considerable fluctuations, the model reached a steady state after 40 ns (Fig. 7c1,c2). The average total potential energies were significantly higher than the other two (Table 7) because of clashes in the model. Superposition of average/initial and average/final conformations showed CA-RMSDs 0.45 nm and 0.3 nm, respectively. However, Fig. 8 shows that the local structures remained almost intact during the simulation time. The CA-RMSD average values for the last 10 ns of the simulation (Table 7) confirmed these findings. The standard deviation (SD) values for the Native and I500T mutant models were 0.01 nm, while the I661T mutant model deviated almost 0.2 nm.

The final models were chosen for further analyses and a brief calculation of percentage similarity between the secondary structures of the native and mutant final conformations were performed in four regions of the AKAP3 protein. These regions are responsible for AKAP3 interactions with different proteins associated with sperm motility and include PKA, AKAP4, and Gα13. The results are summarized in Table 8.

**Table 8:** Calculation of the percentage of similarity between the secondary structures of the native and mutant final conformations after simulation.

Name of the compared models	Percentage similarity of the secondary structures in the desired regions (%)			
	124–143	278–300	488–583	685–853
Native and mutant-I500T final conformation	70	91	87.5	84.1
Native and mutant-I661T final conformation	75	78.3	76.9	87.6

### TM-score assessment results

Based on the TM-score server, none of the mutated models after simulation showed any drastic deviations from the native AKAP3 structure, and all of the TM-score values were within the 0.5–1.00 range (Table 9).

**Table 9:** Assessment of TM-score and RMSD values between the final conformations of the native and mutated AKAP3 structures. *RMSD* root mean square deviation.

Superimposed structures	TM-score	RMSD (Å)
Native-I500T	0.6247	12.051
Native-I661T	0.3220	25.686

## Discussion

AKAPs are considered functionally conserved scaffolding proteins. Some AKAPs mediate important functions in both the male and female reproductive systems and the gametogenesis process^8^.

AKAP3 and AKAP4 have established roles in sperm fibrous sheath assembly during spermatogenesis; their precise coordination is needed for development and maintenance of sperm motility and male fertility. The lack of AKAP3 can lead to global changes in the sperm proteome and mislocalization of sperm proteins^8,14^.

Partial deletions of the AKAP3 and AKAP4 genes that correspond to potential AKAP3/AKAP4 binding sites might be associated with dysplasia of the fibrous sheath (DFS) in some cases^21^. In the present study, we used a combination of in silico approaches to develop a 3D model of AKAP3 and predict the consequences of nsSNPs that experimentally revealed in some infertile men with ISTS and oligoasthenoteratozoospermia.

The ConSurf results for detection of evolutionarily conserved regions of the AKAP3 protein were completely in agreement with experimental surveys^7,10–13^. The conservation degree of an amino acid is strongly correlated with its structural and functional importance. Experimental findings proved that AKAP3 has two helical regions in the N-terminal end (amino acids 124–143 and 278–300 in mice) that are responsible for its interaction with PKA^7^. Amino acids 685–853 at the C-terminal region of AKAP3 also participate in interactions with Gα13^13^. These areas were also characterized as highly conserved by ConSurf, which implies a crucial functional or structural role.

Based on computational analysis, the predicted influences of all amino acid substitutions were interpreted as benign; however, SIFT server indicated that the I661T amino acid was more damaging when compared with the others. Both I500T and E525K are located within a stretch of amino acids that correspond to the creation of a potential AKAP3 binding site for AKAP4 (amino acids 488–583)^12^ whereas, I661T is located out of the AKAP3 binding site for proteins that play a role in sperm motility. According to prediction software outputs, these amino acid replacements are insufficient to disrupt AKAP3 structure and function.

I-Mutant results in terms of AKAP3 stability changes from corresponding amino acid replacements showed that all four amino acid substitutions could lead to a decrease in AKAP3 stability; however, I500T and I661T were more efficient in destabilizing the AKAP3 structure compared to other two changes.

By taking into account the HOPE output, the decrease in AKAP3 stability might be due to differences in physical attributes of nonpolar isoleucine and polar threonine that, in turn, discompose hydrophobic interactions in the protein. Either an increase or a decrease in energy levels of the native protein structure might be associated with altered patterns of protein structure, function, and lead to disease progression^16^; however, destabilization induced by these two conversions seems to be modest.

In 2001, experimental findings by Turner et al. revealed the I500T in both DFS patients and control individuals that was in either the homozygous or heterozygous states. Their observation demonstrated, although this amino acid conversion occurs in a known functional domain of AKAP3 that corresponds to an interaction with AKAP4, no association was observed between the I500T change and DFS, which suggested that either threonine or isoleucine at position 500 of AKAP3 would result in a functional binding domain to AKAP4^12^. Hence, our in silico results for the impact of I500T on the AKAP3 binding site to AKAP4 were entirely in accordance with experimental data.

In order to confirm the in silico investigations, the full-length model of native and mutant AKAP3 were predicted and evaluated as appropriate and reliable structures by Ramachandran plot and the QMEAN score.

Additionally, as PSIPRED prediction is based on the incorporation of two feed-forward neural networks that perform an analysis on output obtained from PSI-BLAST^22^, and DSSP provides the secondary structure based on PDB files^23^, a high percentage of identity in the distribution of secondary structures assigned by using these two servers could also confirm the reliability of the AKAP3 predicted models. Besides, the local structures remained almost intact during the simulation time span in all three models. As the secondary structures that are responsible for the AKAP3 interactions with the PKA, AKAP4 and, Gα13 proteins showed high identities in the final conformation of native and mutant structures, it seems that the I500T and I661T mutations would not have a drastic effect on protein interactions, although more investigation is needed in this regard.

Moreover, the superposition of native and mutant structures by the TM-score server disclosed no drastic deviations, which was in accordance with obtained data.

In silico approaches that use powerful software tools can facilitate predictions of the phenotypic effects of nsSNPs on the physicochemical properties of the proteins of interest. Such information is pivotal for genotype–phenotype correlations and also for recognizing disease biology^24^. Accordingly, as AKAP3 is involved in the emergence of male infertility, providing more structural insights and the impact of nsSNPs that cause protein functional diversity could help researchers elucidate the etiology of this disorder at the molecular level.

Taken together, according to in silico findings, destabilization induced by these substitutions did not appear to cause any considerable structural and functional deviations between the mutated and native AKAP3 that would lead to immotile spermatozoa.

## Conclusion

Collectively, the present study provided a full-length AKAP3 tertiary structure with high degree of quality. This can potentially be a reliable model for further structural studies of the AKAP3 protein. For the first time, we used several bioinformatics approaches to analyze the impact of four nsSNPs on the AKAP3 structure that were present in men with ISTS and oligoasthenoteratozoospermia. Based on our findings, none of the desired variations have the potential to exert any structural impact on the AKAP3 protein. Our computational analysis results in terms of the I500T substitution were completely consistent with previous experimental results. However, the in silico based nsSNP predictions are not solely adequate for deriving genotype–phenotype relations and more experimental investigation is needed to elucidate the impact of the I661T changes on the AKAP3 structure in these patients.

## Materials and methods

### Data mining

Data that pertained to the human AKAP3 gene was retrieved from Entrez Gene on the National Center for Biological Information (NCBI) website. For further computational analysis, information about the desired SNPs and AKAP3 protein sequence were obtained from the NCBI dbSNP (http://www.ncbi.nlm.nih.gov/snp/) and UniProt (https://www.uniprot.org/) databases.

### Assessment of evolutionary conserved regions in AKAP3 using the ConSurf web server

The ConSurf web-server (https://consurf.tau.ac.il/) estimates the evolutionary conservation of amino acids in proteins by using an empirical Bayesian inference. The server analysis is based on phylogenetic relations between homologous sequences. ConSurf output represents conservation scores by a coloring scheme that is subdivided into a distinct scale of nine grades (conservation scores: 1–3 variable; 4–6 average; and 7–9 conserved)^25^. ConSurf also calculates putative functional and structural residues by combining evolutionary conservation findings with solvent accessibility predictions. Highly conserved residues are classified as either functional or structural based on their location on the surface or inside protein^26^.

### Functionally deleterious nsSNPs prediction using a sequence homology-based tool (SIFT)

The conservation level of a distinct position in the protein was assessed by the Sorting Intolerant from Tolerant v.6.2.1 (SIFT) (https://sift.bii.a-star.edu.sg/) server. SIFT applies sequence homology and the physical attributes of amino acids to characterize the functional consequences of nsSNPs on proteins. Substitutions with normalized probabilities less than the tolerance index of 0.05 are predicted to be deleterious or intolerant. Those with more than or equal to the 0.05 index are considered to be tolerated^27^.

### Estimation of the structural and functional effects of nsSNPs by using a structural homology-based prediction server (PolyPhen-2)

Polymorphism Phenotyping v2 (PolyPhen-2) (http://genetics.bwh.harvard.edu/pph2/) is a probabilistic classifier that identifies the possible impacts of amino acid replacements on protein properties by physical and comparative evolutionary considerations. PolyPhen-2 computes the difference between a position-specific independent count (PSIC) score for two variations and determines the likely influence that a single-residue substitution would exert. A mutation is predicted to be qualitatively benign, possibly damaging, or probably damaging based on a probabilistic score (0.0–0.15 benign; 0.15–0.85 possibly damaging; and 0.85–1.0 probably damaging)^28^.

### Simulation of nsSNPs functional consequences using PROVEAN software

Protein Variation Effect Analyzer v.1.1 (PROVEAN) (http://provean.jcvi.org) is a sequence-based predictor that discriminates if a protein’s functional features could be altered by an amino acid substitution. PROVEAN performs a BLAST search to achieve supporting sequences for generation of PROVEAN scores^29^. The threshold of the PROVEAN index is set − 2.5. If the final score of an amino acid change is below the cutoff, it is presumed to be “deleterious”; otherwise, it is classified as “neutral”.

### Project HOPE

Have (y)Our Protein Explained (HOPE) (http://www.cmbi.ru.nl/hope/) is an automatic mutant analysis web application that acquires data from a series of sources such as ‘WHAT IF’ calculations on the PDB-file or homology model built by YASARA, annotations in the UniProt database, HSSP conservation scores, and sequence-based predictions by DAS-servers. HOPE provides a decision scheme for building a report on structural and functional properties exerted by amino acid replacements. This report is illustrated with pictures and animations^30^.

### Characterization of disease-associated amino acids substitutions by MutPred

MutPred2 (http://mutpred.mutdb.org/) is a web application that combines genetic and molecular information to specify the pathogenicity of amino acid replacements and their molecular mechanisms. In fact, MutPred predicts the impact of amino acid changes on more than 50 various protein attributes in order to have efficient inference in the molecular basis of pathogenicity^31^. The FASTA file of the AKAP3 was submitted to the server and amino acid substitutions were entered in the FASTA header. A MutPred general score higher than the 0.5 threshold is interpreted as a pathogenic amino acid substitution.

### Evolutionary analysis of coding SNPs using PANTHER

PANTHER (http://pantherdb.org/) estimates the likelihood of a special nonsynonymous coding SNP to create a functional impact on the protein. It estimates the length of time (in millions of years) a given amino acid has been preserved in the lineage that leads to the protein of interest. The longer the preservation time, the greater the likelihood of a functional impact^32^.

### Computation of protein stability changes upon mutations using I-Mutant2.0 and MUpro

I-Mutant2.0 (http://folding.biofold.org/i-mutant/i-mutant2.0.html) is a support vector machine-based web server trained to disclose stabilization or destabilization of a protein structure induced by the corresponding mutation. I-Mutant2.0 acts as a classifier that elucidates the direction of changes in protein stability due to a mutation, and also as a regression estimator to predict related Gibbs-free energy change (ΔΔ*G*) values^33^. The AKAP3 amino acid sequence was entered in the “protein sequence box” and the numbers of the residues that underwent mutation, as well as the new residues were inserted in the “position and new residue” boxes, respectively. The calculation was conducted at pH 7 and 25 °C. MUpro v.1.0 is a set of machine learning programs that predict how single-site amino acid mutations affect protein stability. A score less than 0 means the mutation decreases the protein’s stability. A smaller score indicates a more confident prediction. Conversely, a score more than 0 means the mutation increases protein stability. The larger the score, the more confident the prediction is (http://mupro.proteomics.ics.uci.edu/).

### Development of a three-dimensional model of AKAP3 via the Robetta server

As The AKAP3 crystal structure was not available in the Protein Data Bank, Robetta (http://new.robetta.org/), which is a comparative modeling or de novo structure prediction server was used. Robetta is an internet service that provides 3D structures for the entire protein sequence in the presence or absence of experimentally determined templates. In the comparative modeling procedure, Robetta infers putative domains based on sequence homology to proteins of known structures; otherwise, it generates models through the Rosetta de novo structure prediction method in the absence of any homology^34^.

The best constructed model from the Robetta server output based on the comparative modeling method was chosen for additional evaluations and model validation. The predicted 3D model of AKAP3 was then visualized using the YASARA v.20.7.4 (http://www.yasara.org/index.html) program. In order to construct the I500T and I661T mutated models, the amino acid substitutions were replaced in the native AKAP3 sequence. The mutants' 3D structures were modeled using the Robetta server and energy minimization was performed.

### Validation of the AKAP3 predicted model

The AKAP3 energy minimized model was validated via the PROCHECK program v.3.0 and the QMEAN server. PROCHECK calculates the stereochemical parameters of the model compared to normal values^35^.

The “degree of nativeness” of the model was also estimated by the Qualitative Model Energy ANalysis (QMEAN) (https://swissmodel.expasy.org/qmean/) server. QMEAN is a composite scoring function that describes the major geometrical aspects of protein structures by using several structural descriptors, including local geometry, the secondary structure-specific distance-dependent pairwise residue-level, and solvation potentials. In comparison to other model quality assessment programs, QMEAN shows a statistically significant improvement over most quality measures that describe the ability of the scoring function to identify the native structure and to distinguish good from bad models^36^.

### Prediction of AKAP3 secondary structure using PSIPRED and DSSP

PSI-BLAST based secondary structure prediction v.4.0 (PSIPERD) (http://bioinf.cs.ucl.ac.uk/psipred/) is a prediction method that produces the distribution of alpha helices, beta-sheets, and coils by analyzing the output obtained from PSI-BLAST^37^. In order to use PSIPRED, the protein sequence was selected as the type of input data and PSIPRED 4.0 was applied as a secondary structure predictor.

Definition of Secondary Structure of Proteins v.2.1.0 (DSSP) (https://swift.cmbi.umcn.nl/gv/dssp/) is the most widely used server for secondary structure assignment. DSSP uses hydrogen bond patterns based on an electrostatic model to assign the secondary structures of all protein entries in the Protein Data Bank^23,38^.

### MD simulation experiment

YASARA suite v.20.7.4 macros^39^ was utilized to run MD simulation experiments and analyze the trajectories. YASARA2 force field^40^ is a built-in force field incorporated in the YASARA suite, which was used to refine the models. The experiments were performed as follows: the hydrogen bonding network was optimized to increase the solute stability^41^; side-chain pKa was predicted for each protein at pH 7.4^42^; and protonation states were accordingly assigned a 0.9% NaCl solution. After energy minimization to remove clashes (the steepest descent and simulated annealing minimizations), each simulation was run using YASARA force field for the solute and TIP3P water model for the solvent. An equilibration time of 250 ps was included in YASARA MD simulation macro with no explicit equilibration; a cutoff of 8 Å for non-bonded real space forces was used to for the most accuracy, even it was time-consuming; and Particle mesh Ewald (PME) were set^43^. We used the algorithms described in detail^40^, and the equations for the motions were integrated with a multiple time-step of 1.25 fs for bonded intra-actions and for non-bonded interactions at a temperature 298 °K and pressure 1 bar (NPT ensemble, as in a living cell). Cell boundaries were set to the periodic boundary. Simulation cells that were 20 A° larger than the proteins were rescaled to reach a density of 0.997 g·l^−1^ for the solvent (water). Each 100 ps a snapshot was saved, in a total 50 ns simulation time span. Of note, a longer simulation time span was not applicable due to our facilities’ limitations. The equilibration time (250 ps) was deleted in the graphs. Carbon-alpha root mean square deviations (CA-RMSDs) were calculated by taking into consideration the initial position. Secondary structures were assigned by the YASARA built-in secondary structure assignment algorithm.

### TM-score calculation

The quantitative assessment of structural similarity between final conformations of the native and mutated models after simulation was measured by the TM-score (https://zhanglab.ccmb.med.umich.edu/TM-score/) server. TM-score is a metric that assesses the topological similarity of protein structures^44^. A TM-score has a value of (0,1], where 1 indicates a perfect match between two structures. Following strict statistics of structures in the PDB, the scores below 0.17 correspond to randomly chosen unrelated proteins and structures that score higher than 0.5 assume generally the same fold in SCOP/CATH.

## Supplementary Information

Supplementary Information.

Supplementary Figure 1.
