# Supplementary material for: Structural modeling of human AKAP3 protein and in silico analysis of single nucleotide polymorphisms associated with sperm motility
Source: Sci Rep. 2022 Mar 7;12:3656. doi: 10.1038/s41598-022-07513-9 (PMC8901789; doi:10.1038/s41598-022-07513-9)
Supplement: Supplementary file 2 — Supplementary Figure 1. [file 41598_2022_7513_MOESM2_ESM.docx]

**Structural Modeling of Human AKAP3 Protein and In-Silico Analysis of Single Nucleotide Polymorphisms Associated with Sperm Motility**

Alemeh Rafaee ^1,2^, Elaheh Kashani-Amin ^3^, Anahita Mohseni Meybodi ^4,5^, Azadeh Ebrahim-Habibi ^3^, Marjan Sabbaghian ^1*^

1. Department of Andrology, Reproductive Biomedicine Research Center, Royan Institute for Reproductive Biomedicine, ACECR, Tehran, Iran

2. Department of Biology, Science and Research Branch, Islamic Azad University, Tehran, Iran

3. Biosensor Research Center, Endocrinology and Metabolism Molecular-Cellular Sciences Institute, Tehran University of Medical Sciences, Tehran, Iran

4. Department of Genetics, Reproductive Biomedicine Research Center, Royan Institute for Reproductive Biomedicine, ACECR, Tehran, Iran

5. Department of Pathology and Laboratory Medicine, Western University, London, Ontario, Canada

*** Corresponding author:** Marjan Sabbaghian

**Address:** No.2, Hafez St, Banihashem St, Resalat Ave, Tehran, Iran

**Tel:** +9821-23562730

**Fax:** +9821-22306481

**E-mail:** [m.sabbaghian@royaninstitute.org](mailto:m.sabbaghian@royaninstitute.org)

Supplementary figure 1


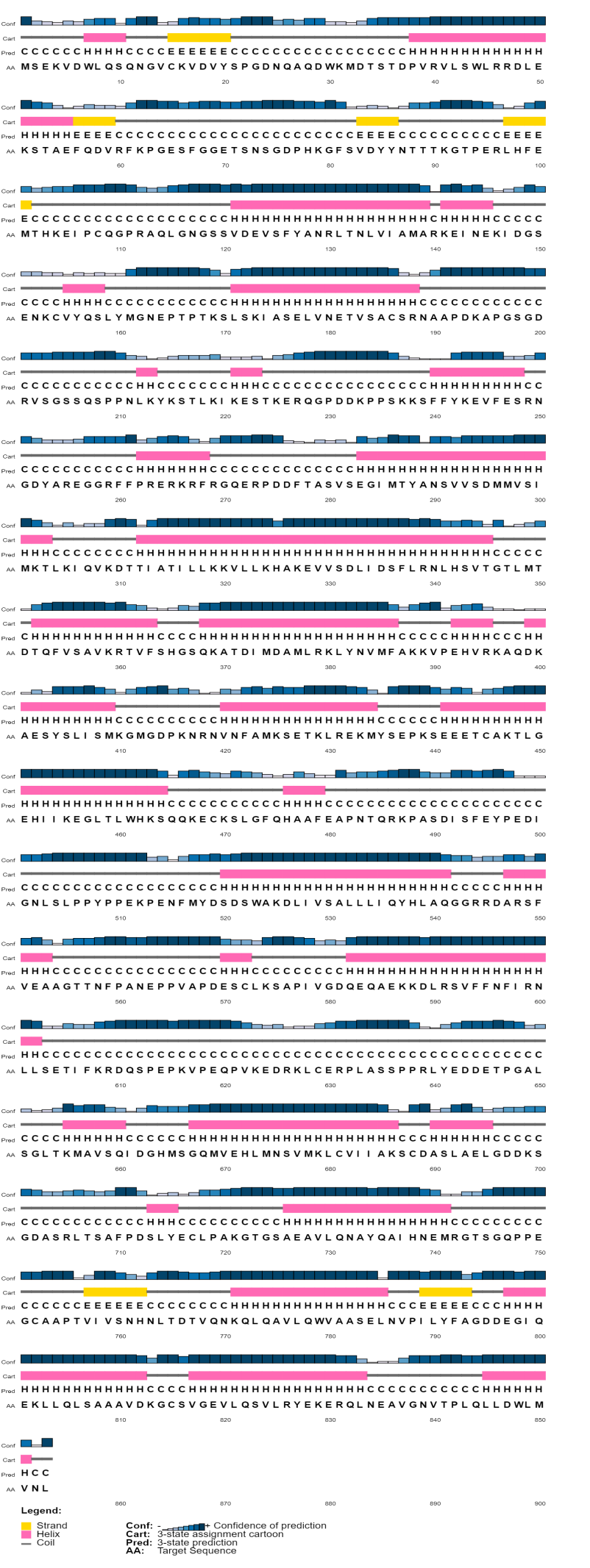
Legend: PSIPRED prediction of the native AKAP3 secondary structures
